# Clinical Characterization of Michelin Tire Baby Syndrome: A Case Report Highlighting Severe Short Stature

**DOI:** 10.7759/cureus.72345

**Published:** 2024-10-25

**Authors:** Kenji Iwai, Manabu Okawada

**Affiliations:** 1 Pediatric Department, Sunrise Japan Hospital Phnom Penh, Phnom Penh, KHM

**Keywords:** complication, follow-up, kunze-riehm syndrome, michelin tire baby syndrome, short stature

## Abstract

Kunze-Riehm syndrome, commonly known as Michelin tire baby syndrome (MTBS), is a rare genetic disease characterized by multiple circumferential skin folds on the limbs and trunk. The exact etiology, mechanism, and prognosis of MTBS remain poorly understood. Symptoms other than the circumferential skin folds, as well as the severity of the condition, vary among cases, ranging from only skin folds to severe neurological complications. Herein, we report a four-year-old Cambodian girl with no past medical history who presented with significant short stature. She exhibited characteristic circumferential skin folds on her limbs, hypertelorism, and a sunken nasal bridge. On her first visit, her height was only 74.5 cm (-6.5 SD). Her mental development was appropriate for her age, and none of her family members exhibited similar symptoms. Over four years of follow-up, the patient showed gradual physical development, reaching a height of 95.9 cm (-5.4 SD) at the age of eight. Physicians must recognize that patients with MTBS can present with significant short stature as part of their symptoms. Additionally, although the diagnosis of MTBS might be delayed in low- and middle-income countries, including Cambodia, possibly due to ambiguous diagnostic criteria and limited access to medical care, regular follow-up is crucial for patients with MTBS due to potential complications and the uncertainty of prognosis.

## Introduction

Kunze-Riehm syndrome, commonly known as Michelin tire baby syndrome (MTBS), is a rare genetic disease characterized by multiple skin folds on the limbs and trunk. The name MTBS originates from the resemblance to the mascot of a French company [1]. However, there have been suggestions to adopt a new name and establish more accurate diagnostic criteria due to the potentially insulting nature of the term "MTBS" and the ambiguity of its diagnosis [2]. Although there are some studies on specific gene expressions [3], the exact etiology, mechanism, and prognosis of MTBS remain poorly understood. Additionally, the severity and complications vary significantly among cases. However, MTBS is associated with significant short stature and its long-term development has not been well-documented. Herein, we report a patient diagnosed with MTBS at the age of four, presenting with significant short stature, who subsequently showed gradual development over four years of follow-up.

## Case presentation

**Figure 1:**
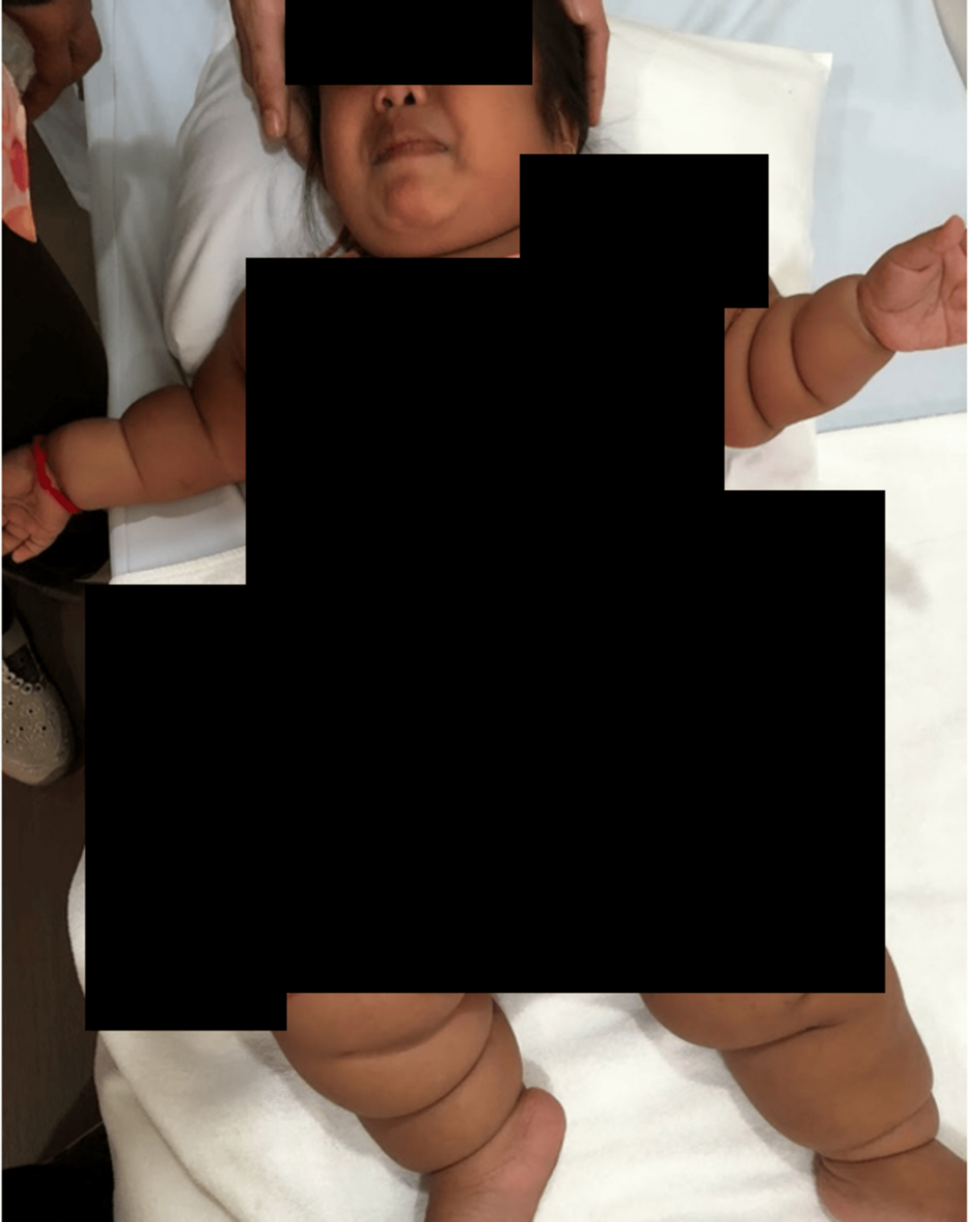
Circumferential skin creases on both sides of her limbs

A four-year-old Cambodian girl with no past medical history presented to the pediatric department of Sunrise Japan Hospital Phnom Penh with chief complaints of snoring, short stature, and being overweight. Physical examination revealed circumferential skin creases on both sides of her limbs (Figure 1).

**Figure 2:**
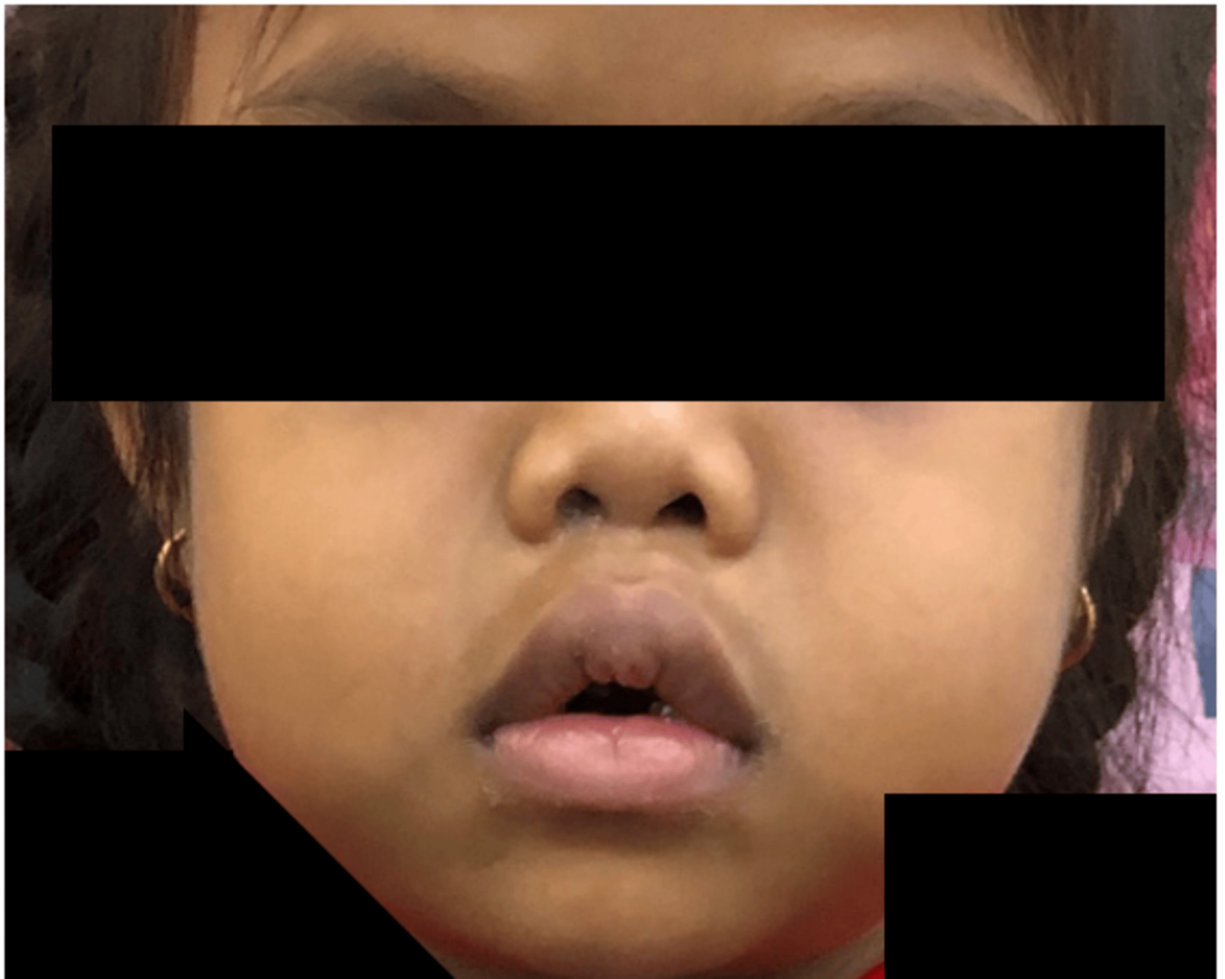
Hypertelorism and a sunken nasal bridge

She had a short neck and characteristic facial features, including hypertelorism and a sunken nasal bridge (Figure 2).

**Figure 3:**
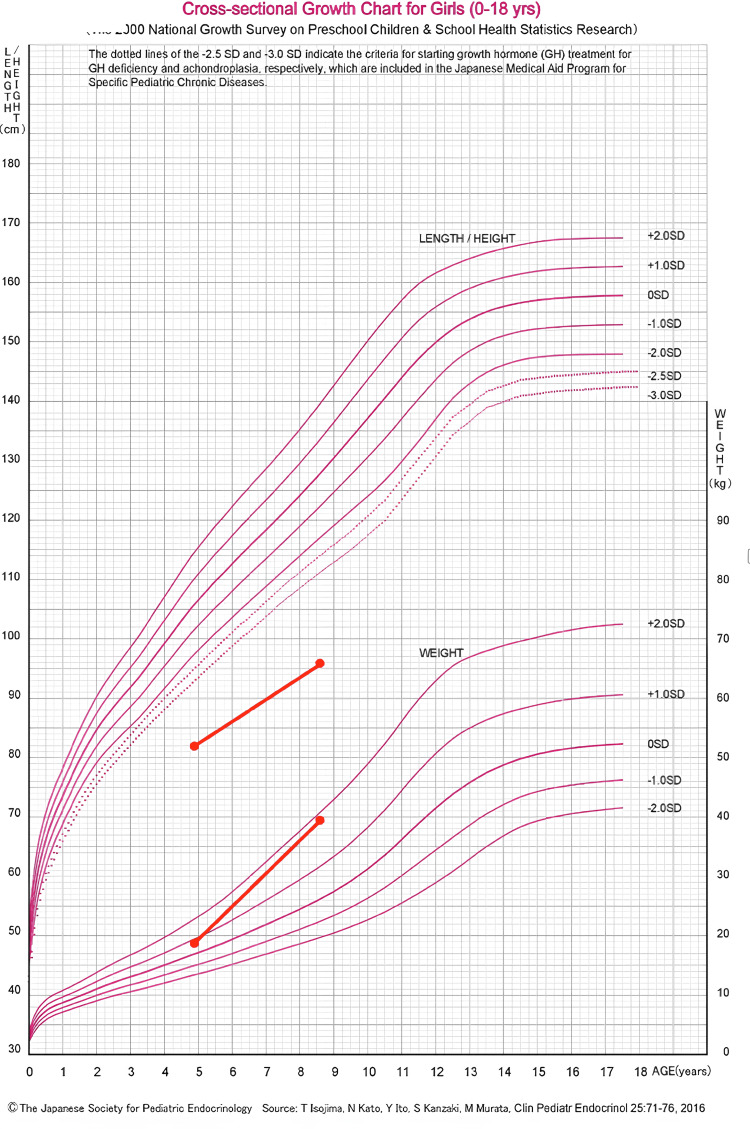
The growth chart of the patient

Her tonsils were enlarged to grade three, causing severe snoring. We educated her and her parents on maintaining an appropriate airway position at night and controlling her body weight through a strict diet and physical training. She exhibited significant short stature, measuring 74.5 cm (-6.5 SD), and was overweight at 24 kg (+2.9 SD). The patient was born full term with a birth weight of 3,100 g via standard vaginal delivery and experienced no events during the antenatal period. She had no family history of similar conditions. Her insulin-like growth factor (IGF-1) levels were within the normal range at 206 ng/mL, and her karyotype was 46XX, indicating a normal female. Thyroid function was within the normal range. A skin biopsy was not performed due to a lack of resources. Her mental development was appropriate for her age. The diagnosis of MTBS was made clinically based on physical findings. After over four years of follow-up without any interventions, her height reached 95.9 cm (-5.4 SD) at the age of eight (Figure 3).

Although the skin folds remain unchanged and her height is still significantly shorter than the normal range for her age, the patient showed gradual physical development with no other complications.

## Discussion

We described a four-year-old case of MTBS with significant short stature who subsequently showed gradual physical development over four years of follow-up without any other complications. In this case, we learned two important things regarding MTBS: the possibility of significant short stature as a complication and the importance of regular follow-up even when the diagnosis is delayed.

Regarding the first clinical point, we found that patients with MTBS can exhibit significant short stature as the main symptom. The severity and complications vary significantly among cases. The most common complications reported include facial anomalies, such as malformations of the auricles of the ear, broad or sunken nasal bridge, epicanthic folds, hypertelorism, cleft palate, microphthalmos, micrognathia, and central facial flattening [4]. Several case studies have shown that patients with various complications, such as significant developmental delay [2,5], achondroplasia [6], hydrocephalus [2], panhypopituitarism [7], and hypotonia [8], are accompanied by characteristic skin creases from an early stage of life. Conversely, skin folds have been reported to naturally resolve over time [9]. In this case, the main problem other than circumferential skin folds on the limbs and facial anomalies was short stature. For the differential diagnosis of significant short stature, skeletal dysplasias such as achondroplasia, hypochondroplasia, and thanatophoric dysplasia can be considered. Since the diagnosis of MTBS largely depends on clinical findings, possible ambiguity remains as to whether the skin folds are caused by shortened limbs and excess skin due to osteogenesis imperfecta or MTBS. Among skeletal dysplasias, thanatophoric dysplasia has been reported to present multiple skin folds on the limbs; however, this case did not show any clinical symptoms suggesting thanatophoric dysplasia, including fatal respiratory failure [10,11]. Although a case report mentioning short stature in MTBS has been published [12], no case reports specifically focusing on short stature have been published in English to date.

Regarding the second clinical point, patients with MTBS should be followed up long term even when the diagnosis is delayed. One reason for the delayed diagnosis in this case could be the ambiguity of the diagnostic criteria. Although it has not yet been established, some authors have suggested new diagnostic criteria and a new disease name instead of MTBS due to its potentially insulting nature [2] and ambiguity [3]. A skin biopsy showing either smooth muscle hamartoma, nevus lipomatous, or degenerative collagen might aid in diagnosing MTBS [3,4]. However, in many cases, the diagnosis is made without a skin biopsy, and we could not perform it due to a lack of resources and funds. Chromosomal abnormalities, such as deletion of chromosome 11 [13] and inversion of chromosome 7 [14], have been associated with MTBS, but normal chromosomes do not rule out the condition [3]. In this case, the diagnosis was finally made clinically at the age of four despite significant short stature. Fortunately, the patient did not show any further complications and exhibited physical development over four years of follow-up. MTBS can present a wide variety of complications [3]. Even when the diagnosis is delayed for some reason, patients with MTBS should be regularly followed up due to the potentially unclear prognosis and complications.

## Conclusions

In conclusion, we must acknowledge that patients with MTBS have the possibility of significant short stature as part of a variety of symptoms. Due to the ambiguity of the diagnosis for MTBS, other differential diagnoses should be carefully considered. Additionally, it is necessary to closely follow up with patients with MTBS, even when the diagnosis is delayed for any reason.
